# Significance of Persistent Inflammation in Respiratory Disorders Induced by Nanoparticles

**DOI:** 10.1155/2014/962871

**Published:** 2014-07-07

**Authors:** Yasuo Morimoto, Hiroto Izumi, Etsushi Kuroda

**Affiliations:** ^1^Institute of Industrial Ecological Sciences, University of Occupational and Environmental Health, Japan; ^2^Laboratory of Vaccine Science, WPI Immunology Frontier Research Center, Osaka University, Japan

## Abstract

Pulmonary inflammation, especially persistent inflammation, has been found to play a key role in respiratory disorders induced by nanoparticles in animal models. In inhalation studies and instillation studies of nanomaterials, persistent inflammation is composed of neutrophils and alveolar macrophages, and its pathogenesis is related to chemokines such as the cytokine-induced neutrophil chemoattractant (CINC) family and macrophage inflammatory protein-1*α* and oxidant stress-related genes such as heme oxygenase-1 (HO-1). DNA damages occur chemically or physically by nanomaterials. Chemical and physical damage are associated with point mutation by free radicals and double strand brake, respectively. The failure of DNA repair and accumulation of mutations might occur when inflammation is prolonged, and finally normal cells could become malignant. These free radicals can not only damage cells but also induce signaling molecules containing immunoreaction. Nanoparticles and asbestos also induce the production of free radicals. In allergic responses, nanoparticles act as Th2 adjuvants to activate Th2 immune responses such as activation of eosinophil and induction of IgE. Taken together, the presence of persistent inflammation may contribute to the pathogenesis of a variety of diseases induced by nanomaterials.

## 1. Persistent Inflammation and Harmful Effects

Reports on the toxicology of nanomaterials have been increasing recently, but the effect of nanomaterials on the human body is inconclusive. It is thought that, in general, inhaled dusts such as particles and fibrous materials in the lung repeatedly induce inflammation and finally lead to pulmonary fibrosis and respiratory cancer [1, 2]. It is considered that the presence of persistent inflammation leads to advanced stages such as fibrosis and tumors. Persistent inflammation, reported in animal exposure models using asbestos and silica, is important in the pathology of the formation of irreversible chronic lesions [1, 2]. In an examination of inhalation exposure of rat to chrysotile for 20 days, continuous inflammation and fibrosis containing mainly neutrophils were observed [3]. Intratracheal instillation of crystalline silica induced a persistent neutrophil inflammation in rat lung. This inflammation progressed time-dependently during 6 months after exposure [4]. Pulmonary persistent inflammation is also thought to be related to lung disorders induced by manufactured nanomaterials. Among nanoparticles, nickel oxide nanoparticles, a material with high toxicity, induced persistent inflammation in the lung [5, 6]. Nishi et al. [6] reported that nanoparticles of nickel oxides induced persistent neutrophil inflammation in the rat lung from 3 days to 3 months after intratracheal instillation. There are many reports that carbon nanotubes induced persistent inflammation in rats and mice after intratracheal instillation or inhalation.

In order to examine what kinds of cytokines are related to lung disorders induced by nanoparticles, Morimoto et al. measured the concentrations of 27 cytokines including inflammation, fibrosis, and allergy-related ones, in the lung and bronchoalveolar lavage fluid (BALF) following intratracheal instillation of well-dispersed nickel oxide nanoparticles [5]. The expression of macrophage inflammatory protein-1*α* (MIP-1*α*), heme oxygenase-1 (HO-1), cytokine-induced neutrophil chemoattractant-1(CINC-1), and CINC-2 showed a continued increase in the lung tissue and BALF, while interleukin-1*α* (IL-1*α*), IL-1*β* in the lung tissue, and monocyte chemotactic protein-1 (MCP-1) in BALF showed transient increases. In another experiment, Fujita et al. [7] analyzed the comprehensive gene expression by microarrays and found that CINC-1, 2, MIP-1, HO-1, and matrix metalloproteinase-12 (Mmp-12) expressions increased with exposure to nickel oxide nanoparticles, while nearly no increase of other neutrophil chemokines was observed. This persistent expression of the CINC family suggests that chemokines are important in neutrophil inflammation in lung exposed to nanoparticles. Diesel particles [8], inhaled materials with inflammatory potentials, have been reported to persistently increase CINC-1 or CINC-2 expression in the lung following intratracheal instillation. On the other hand, TiO_2_ (micron-size) and fullerene, which are less inflammogenic to the lung, revealed a mild and transient increase in CINC-1 and CINC-2*α* *β* expression only at an acute phase after intratracheal instillation [9]. Nickel oxide nanoparticles induced only a transient expression of CINC-3 in an intratracheal instillation study, although the nickel oxide nanoparticles induced persistent pulmonary inflammation in the rat lung [6, 10]. In that intratracheal instillation study, the maximum dose of nickel oxide nanoparticles was 0.2 mg/rat. We performed an intratracheal instillation study with a high dose, and 1 mg of nickel oxide nanoparticle induced a persistent increase in CINC-3 concentration and more severe neutrophil inflammation in rat lung. Taken together, we suspect that CINC-3 may play a role in enhancing pulmonary inflammation. There is a report [11] that a difference in biological activities of the CXC chemokine receptor 2 was observed between CINC-1, CINC-2, and CINC-3. CINC-3 induced the enhancement of cytoplasmic calcium concentration more potently than did CINC-1 and CINC-2 in the short-term incubation (<10 min) of bovine alveolar macrophage with quartz dust particles, a material with inflammatory potential.

If the high dose of nanomaterials was increased too much, even particles with low toxicity induced persistent inflammation, fibrosis, and tumor in the lung following not only intratracheal instillation but also inhalation. A 2-year inhalation exposure study of TiO_2_ micron-sized particles, which are considered to be negative control, at concentrations of 0, 10, 50, and 250 mg/m^3^ was conducted on rats, and bronchioloalveolar adenomas and cystic keratinizing squamous cell carcinomas occurred at 250 mg/m^3^ exposure, while no compound-related lung tumors were found in rats exposed to either 10 or 50 mg/m^3^ [12]. These data suggested that an overload of titanium dioxide, in spite of its toxicity, may induce lung tumors at high dose exposure. The overload of materials is due to a dysfunction of alveolar macrophage, and this phenomenon is accompanied by a delay of clearance of materials from the lung and the pulmonary response. As for nanoparticles, Bermudez et al. [13] performed a subchronic inhalation (13 weeks) of ultrafine TiO_2_ particles at aerosol concentrations of 0.5, 2.0, and 10 mg/m^3^. Exposure to 10 mg/m^3^ induced the retardation of particle clearance and neutrophil infiltration in rat lung; on the other hand, exposure to 2 mg/m^3^ caused neither reaction. The inflammation at 10 mg/m^3^ was accompanied by delay of clearance, and the inflammation induced by the titanium dioxide nanoparticles may have been due to an overload of titanium dioxide. We speculate that the difference in deposition rate of nano- and micron-sized particles of TiO_2_ (the amount of deposition of nanoparticles in the lung is more than that of micron-sized particles) may affect only nanoparticle to induce the pulmonary response in the lung in spite of same concentration (10 mg/m^3^). Therefore, measurements of the harmful effect of nanoparticles may be performed using data from concentrations which are below the dose of overload, such as equal to or less than 2 mg/m^3^ titanium dioxide nanoparticles.

**Figure 1:**
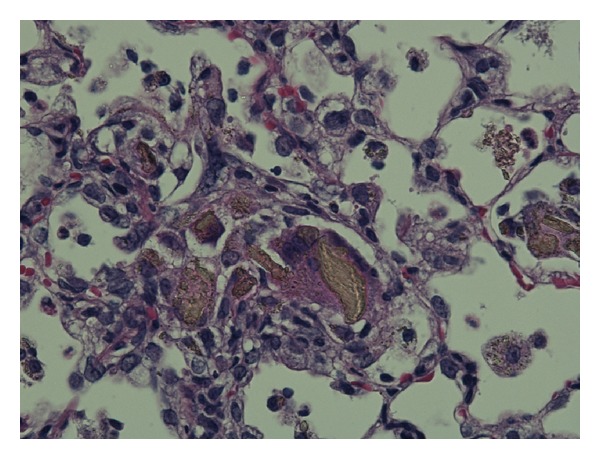
Hematoxylin and eosin staining of lung sections exposed to 3 mg TiO_2_ nanoparticles at 3 days after instillation.

In cases of intratracheal instillation of particles, an excess dose induces the artificial effect of the bolus. We conducted an intratracheal instillation of 3 mg/rat of titanium dioxide nanoparticles [14].  Figure 1 shows a large granulomatous lesion including local accumulation of TiO_2_ nanoparticles in the bronchoalveolar area 3 days after exposure. These lesions in the lung are not seen in usual inhalation studies. From this point of view, exposure of animals to excess doses of nanoparticles should be avoided. Although the most suitable dose of nanoparticles for the evaluation of harmful effects is not known, 0.2 mg/rat (0.67 mg/kg) of nickel oxide nanoparticles with high toxicity induced persistent neutrophil inflammation in rats [6, 15], and 1 mg (3.3–5 mg/kg) of fullerene and titanium dioxide nanoparticles with low toxicity induced transient inflammation [9, 14, 16]. If the relative harmful effect between nanoparticles is measured under the same weight base, pulmonary responses at doses from 0.2 mg/rat to 1 mg/rat (0.67–5 mg/kg) may be useful, at least partially.

It is important to estimate how long the persistent inflammation in an animal model is related to the toxicity of the nanoparticles. From previous studies, we think that 3 or 6 months of persistent inflammation from the end of exposure is related to a high or medium toxicity of nanoparticles [17].

We performed intratracheal instillations of different mineral fibers to rats and examined lung inflammation from 3 days up to 6 months [17]. Harmful respirable particles like crystalline silica or crocidolite asbestos, which are kinds of asbestos, caused persistent inflammation from the initial instillation until 6 months later. However, when less harmful micron-sized titanium dioxide was inhaled, transient inflammation was only observed early in the instillation. As for crystalline silica, an exacerbation of inflammation was found in the lung after 1 or 2 months after exposure. Sellamuthu et al. [18] reported that when rats were exposed to inhalation of crystalline silica (15 mg/m^3^, 6 h/day, 5 days), pulmonary damage was determined after the latent periods (0–16 weeks). The number of neutrophils and the concentration of MCP-1 in BALF were maximum after 16 weeks. Langley et al. [19] conducted a 6-week inhalation of silica with 27 weeks after exposure, and the number of neutrophils and lymphocytes in BALF increased 10 weeks after exposure, although not at 4 days, and LDH and protein concentration in BALF significantly increased at 10 and 17 weeks, but not at 4 days. Kobayashi et al. [16] showed that different evaluations of pulmonary toxicity by intratracheal instillation of titanium dioxide nanoparticles can be derived on the basis of observations up to 1 week after instillation and those after 1 month after instillation. Based on the results of intratracheal instillation studies and inhalation studies, both short-and long-term effects (from 3 days up to 6 months) should be evaluated when assessing the toxicity, including persistent inflammation, of nanoparticles. Therefore we speculate that exposure of the high toxic nanomaterial may induce persistent inflammation in the lung through the persistent production of chemokines, such as CINC, MIP, and MCP, and that sustained production of proteinases and ROS cause the lung injury during these chronic inflammations. Fujita et al. [7] reported that exposure to nickel oxide nanoparticle following intratracheal instillation induced persistent proteinases such as MMP-12 in rat lung.

## 2. Relationship between Inflammation and Malignant Tumor (Figure 2)

Malignant tumor is a polygenic abnormality disease caused by the accumulation of mutations in the genome of a normal cell, such as by single nucleotide substitution, deletion or insertion of a nucleotide, or translocation. Hanahan and Weinberg reported that malignant tumors should acquire six biological properties: self-sufficiency in growth signals, insensitivity to growth-inhibitory (antigrowth) signals, evasion of programmed cell death (apoptosis), limitless replicative potential, sustained angiogenesis, and tissue invasion and metastasis [20]. In 2011, four other biological properties (deregulation of cellular energy, avoidance of immune destruction, genome instability, and mutation and tumor-promoting inflammation) were added [21]. These properties are obtained when gene mutations accumulate. For the accumulation of genetic mutations, it is necessary to argue from two viewpoints: DNA damage and DNA repair. When DNA damage is caused by asbestos and nanomaterials containing nanoparticles and nanofibers as well as mutagens, and the damage cannot be repaired, genetic mutations accumulate and malignant tumors can occur. Interestingly, Xu et al. reported that multiwalled carbon nanotubes are similar to asbestos and have higher risk of causing asbestos-like pleural lesions [22]. For that reason, not only nanomaterials but also asbestos is reviewed.

There are two kinds of mutations in tumors: passenger mutations and oncogenic driver mutations [23]. Passenger mutation occurs only by chance, and oncogenic driver mutation occurs in important genes involved in the phenotype of cancer. Oncogenic driver mutations contain EGF receptor, K-ras, HER2, AKT1, and so forth [24–26]. In addition, there is a cancer that is completely dependent on the oncogenic signal associated with cell proliferation, and survival of cancer by only one mutated gene is possible [27]. This state is called oncogene addiction, and a representative example is L858R mutation in the EGFR gene.

It is known that exposure to asbestos and nanomaterials induces cell dysfunctions at various levels, such as cell death by oxidative stress, DNA damage, and protein damage. DNA damage is associated with malignant tumors and is closely related to inflammation. Nanomaterials in inhalation or intratracheal instillation can cause acute and chronic inflammation to the respiratory tract and pulmonary alveolar space [28, 29]. In particular, persistent inflammation causes fibrosis of the lung and pleura and progresses to lung cancer or malignant mesothelioma [30]. Persistent inflammation causes lung damage, in which the production of free radicals due to inflammation is the most important cause. There are two types of free radicals, reactive oxygen species and reactive nitrogen species [31]. In the reactive oxygen species, there are superoxide ion (O_2_^−^), hydroxyl radicals (^•^OH), hydrogen peroxide (H_2_O_2_), and singlet oxygen (^1^O_2_). ^•^OH is the most reactive of these reactive oxygen species. On the other hand, in the reactive nitrogen species, there are nitric oxide (NO), nitrosonium ion (NO^+^), nitrite ion (NO_2_^−^), and peroxynitrite (ONOO^−^). ONOO^−^ is the most reactive of these reactive nitrogen species. Free radicals are produced spontaneously in the energy metabolism of the cell. Asbestos and nanomaterials are taken into the body, and free radicals are produced on their surface by inflammatory cells or epithelial cells phagocytizing them. TiO_2_ [32], asbestos, and silica [33] have been reported as examples of radicals produced on the surface of asbestos and nanomaterials, while phagocytic cells such as neutrophils and macrophages play a role essential to the host defense to produce superoxides by the active oxygen production enzyme system, such as NADPH oxidase [34]. 8-hydroxydeoxyguanosine (8-OHdG) and 8-nitroguanine (8-NG) are known as DNA damages caused by reactive oxygen and nitrogen species, respectively [35, 36]. It has been reported that nanomaterials cause these damages [37–39]. It was reported that reactive oxygen species generated by titanium expressed Fas, Bcl-2-associated X protein (Bax), IL-1 beta, and induced apoptosis [40, 41]; those generated by silica induced DNA damage and autophagy [42, 43], those generated by polyvinylpyrrolidone (PVP)-coated silver nanoparticles and silver ions induce apoptosis and necrosis in THP-1 monocytes [44], and those generated by carbon nanotube activated p38 MAPK and NF-*κ*B signaling and induced fibrogenic and angiogenic responses [45–47].

Mutations of tumor protein p53 (TP53), Kirsten rat sarcoma viral oncogene homolog (K-ras), epidermal growth factor receptor (EGFR), and neurofibromatosis 2 (NF2) caused by asbestos and nanomaterials are frequently reported. K-ras mutation plays an important role in signal transduction of EGFR. Both K-ras and EGFR mutations are oncogenic driver mutations. Andujar et al. [48] reported that TP53, EGFR, and K-ras mutations were found in non-small-cell lung cancer without association with asbestos exposure and that NF2 was only altered in MPM. Nelson et al. [49] reported that asbestos exposure increases the mutation at K-ras codon 12 and that this process occurs independently of the induction of interstitial fibrosis. Husgafvel-Pursiainen et al. reported that asbestos exposure alone was not significantly associated with an increased occurrence of K-ras mutations. However, a strong and significant association was found between adenocarcinoma and K-ras mutation in a group of smokers with asbestos exposure [50, 51]. Recently, Shvedova et al. reported that single-wall carbon nanotube and carbon nanofibers, but not asbestos exposures, increased the incidence of K-ras oncogene mutations in the lung [52]. However, there are few reports associated with nanoparticles and oncogene mutation. To investigate the occurrence of oncogene mutations with long exposure of nanoparticles in lung is required.

Free radicals can not only damage cells but also induce signaling molecules containing immunoreaction [53], remodeling of the extracellular matrix [54, 55], regulation of cell proliferation [56], and malignant transformation [57].

DNA damage caused by free radicals is an indirect damage caused by asbestos and nanomaterials. On the other hand, low soluble nanomaterials that are phagocytosed may contact DNA directly. Mu et al. reported that multiwalled carbon nanotubes (MWCNTs) could perform nuclear translocation [58]. This result suggests that the fibers could induce DNA damage directly. But it is not clear whether the penetration of the nuclear membrane by MWCNTs is active or passive. It may be possible that nanomaterials contact DNA directly even in the cytoplasm, because the nuclear membrane disappears in the chromosomal distribution in cell division. DNA cleavage is one of the predicted DNA damages caused by contact with DNA. Translocation may occur if DNA cleavage is not repaired correctly. The occurrence of malignant tumors by translocation has been reported in various leukemias [59]. It was recently reported that genomic translocation in lung cancer was observed and that the echinoderm microtubule-associated protein-like 4-anaplastic lymphoma kinase (EML4-ALK) fusion gene plays an important role in lung cancer [60]. However, it is not known how the EML4-ALK fusion gene occurs.

**Figure 2:**
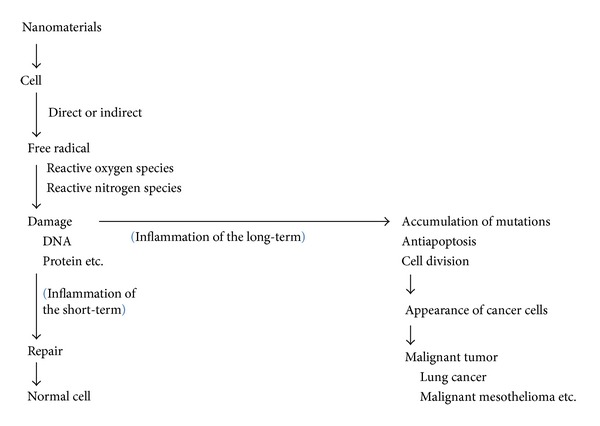
Tentative relationship between inflammation by nanomaterials and malignant tumor.

It is conceivable that gene mutations accumulate when repair is not in time for a lot of DNA damages by nanomaterials or when nanomaterials are not cleared because of their physical and chemical properties. Various pathogenic substances cause acidic conditions around the inflammation [61]. Various proteins forming a complex work in the DNA repair system, including nucleotide excision repair, base excision repair, mismatch repair, homologous recombination, and nonhomologous end-joining [62]. It is thought that an acidic condition inhibits the complex formation of proteins working on DNA damage recognition and DNA repair. Yuan et al. [63] reported that DNA repair was diminished and mutagenesis was elevated in mammalian cells by exposure of hypoxia and low pH. Therefore, gene mutations might accumulate easily by asbestos and nanomaterials where inflammation is sustained (Figure 2). Further research is needed to determine whether persistence of inflammation is a cause of cancer.

## 3. Allergic Response

It has been revealed that the presence of inflammation plays a key role in the formation of allergic disease. It is thought that persistent inflammation, especially allergic inflammation, is related to the onset and progression of allergic disease. In bronchial asthma, persistent allergic inflammation induces airway remodeling, including deposition of collagen, and progresses to intractable asthma suggesting that sustained allergic inflammation contributes to the pathogenesis of the disease [64–67].

Particle materials, unlike allergens, are reported to have properties to enhance the immune response against antigens, the so-called adjuvant effect, and act as Th2 adjuvants to activate Th2 immune responses such as activation of eosinophil and induction of IgE [68]. There are reports that crystalline silica, nickel oxide nanoparticles, and carbon nanotubes induced IgG1 and IgE [69, 70]. Because nanoparticles and fibrous materials also induce Th2 immune responses, the surface area and length of fibrous materials may affect not only nonspecific responses but also immune-specific responses. The molecular and immunological mechanisms of action of particles in immune responses are poorly understood. Almost all particles preferentially induce Th2 immune responses; therefore it has been hypothesized that the specific signals evoked by particles in immune cells are involved in triggering in Th2 immune responses.

In 2008, several reports focused on the discovery that particles activate the NLRP3 inflammasome [71, 72]. The inflammasome is one of the pattern recognition receptors and is expressed in intracellular. The NLRP3 inflammasome is activated by particles such as aluminum salts, crystalline silica, and asbestos. Activated NLRP3 inflammasome promotes the production of IL-1*β* and IL-18, and these cytokines are considered to be involved in the induction of immune responses.

Some particles have cytotoxic activities and induce cell death. Dead cell-derived factors, what we call damage-associated molecular patterns (DAMPs), are known to stimulate immune cells. Uric acid is a purine catabolite that is released from dying cells. It is reported that uric acid or monosodium urate crystal (MSU) stimulates immune cells [73]. In addition, uric acid is released at the site of administration of aluminum particle [73].

Not only uric acid, it is also reported that the DNA released from damaged cells mediates the adjuvant activity of particles. Released host DNA is considered to be recognized by intracellular DNA sensors, but the detailed mechanisms by which the host DNA triggers the immune responses are unclear. Several reports have shown that stimulator of interferon genes (STING), interferon regulatory factor 3 (IRF3), and TANK-binding kinase 1 (TBK1), which are molecules associated with the signal pathway activated by host DNA, is required for the adjuvant activity of aluminum particle [74, 75].

Another unique mechanism is reported that lipid mediator, prostaglandin E_2_ (PGE_2_), is released by macrophages in response to particles and activates immune responses. PGE_2_ is the well-characterized proinflammatory lipid mediator synthesized from arachidonic acid. It is reported that PGE_2_ released by particles stimulates B cells to induce IgE [70].

Many factors are likely to be involved in the adjuvant activity of particles, and a consistent mechanism has not been found. We expect to elucidate what kinds of factors are involved in persistent allergic inflammation.
